# Public health and politics: how political science can help us move forward

**DOI:** 10.1093/eurpub/cky194

**Published:** 2018-11-01

**Authors:** Marleen P M Bekker, Scott L Greer, Natasha Azzopardi-Muscat, Martin McKee

**Affiliations:** 1 Department of Social Sciences, Chair Group Health and Society, Wageningen University and Research, Wageningen, The Netherlands; 2 Health Management and Policy, University of Michigan School of Public Health, Ann Arbor, MI, USA; 3 Department of Health Services Management, University of Malta, Msida, Malta; 4 European Public Health, London School of Hygiene and Tropical Medicine, London, UK

Public health and politics are two sides of the same coin. Just combining the words ‘public’ and ‘health’ makes a clear statement that health can only be achieved by the concerted action of many people who must work together in pursuit of a common goal. Acknowledging this raises questions, though: How should they work together? As a voluntary grouping of those with shared interests, where people can join or leave as they desire? Or within an organized state, governed by laws that safeguard rights but demand obligations? Such questions, addressing issues like the relationship between the individual and the state and the distribution of power and resources in society, are at the heart of political science. But they are also crucial to efforts to improve health. Too often, we shy away from politics, instead adopting a narrow and, arguably, easier technocratic approach, setting out why an evidence-based action should be done without asking how it might be done and devising an appropriate implementation strategy.

This Special Issue is an initiative by EUPHA’s Public Health Practice and Policy section. Taking as a motto: that ‘the philosophers have only interpreted the world, in various ways. The point, however, is to change it’,^1^ it responds to the call ‘for public health to extend its rigorous analysis to politics, drawing and building on the insights of political science to fulfil the promise of public health that it strives to cure society’s ills, rather than just diagnose them’.^2^ Recalling writings that go back to Virchow,^3^ who attributed the deaths during the 19th century typhus epidemic in Silesia to the power of the aristocracy supported by the church and, more recently, by Bambra et al.^4^ and De Leeuw et al.^5^ among others, they call on the public health community to ask what shapes the political options and techniques for public health, and when or not do we use them given the strategic landscapes of the political systems we navigate in?

The papers in this Special Issue seek to address these prescriptive questions by taking an empirical approach to studying how it is actually practiced and with what gains or consequences. It describes a variety of middle range theories on how different arrangements for ‘puzzling’ and ‘powering’ can offer an improved understanding of politics in some key areas for public health. Each paper describes a political science concept illustrated by a key public health issue. Inevitably, it has not been possible to cover the entire range of political science as it relates to health. Systematic comparisons, for instance, of the politics of different public health issues at a variety of scales in government and society could further deepen our understanding that there is no ‘one-size fits all’ approach and public health political strategy needs tailoring to specific circumstances and positions of actors.

This series is especially timely when political disruptions cause uncertainty and risk on many issues including health, and, more specifically, when the Italian government has reversed the decision of its predecessor to make childhood immunization mandatory, and when an Austrian government has abandoned plans by its predecessor to impose a smoking ban in public places. In Brexit, the United Kingdom is pursuing a policy that will cause profound damage to its health system, also putting lives at risk.^6^ In all of these cases, the public health community has assembled and presented the evidence, but without success. As political configurations move away from the mainstream politics of the 20th century, with ‘fringe’ movements becoming parties in government and mainstream parties changing their stance and priorities, these ‘Winds of Change’ in Europe inevitably require us to reconsider our approach to public health politics.

The European Public Health Association, by supporting this Special Issue has chosen to start this important debate. With this Issue we hope to show you some of the opportunities beside the threats of politics, and the assets that make our democracy work (sometimes, even if it is rare and severely under pressure). Politics has a very bad name but it has much to offer if we know how it works. A solid understanding makes us more capable of professionally -that is informed by political science–organizing supportive structures that coordinate public health values and priorities with other values and priorities in society (meaning that we urgently need to move beyond the public health community into the wider and especially the ‘hostile’ environment exploring and building new coalitions). Secondly, having strong enduring strategies in place, such as professional public affairs processes, enables us to add to our evidence base strategic intelligence about stakeholders’ agendas and moves, organizing a receptive environment for the solid science we already have in place. This requires a flexible and pragmatic mindset in parts of the public health workforce, while safeguarding the authority of specialist expertise in other areas. With these investments we are empowering ourselves to exert effective influence, and empowering others to take smart and responsive decisions. This Issue, with each paper offering an extensive biblio-graphy for a political evidence base, offers a first step in that direction.

## Funding

This open access Supplement received funding under an operating grant from the European Union's Health Programme (2014–2020).

*Conflicts of interest*: None declared.
